# Nutritional and Healthy Value of Chemical Constituents Obtained from Patagonian Squid (*Doryteuthis gahi*) By-Products Captured at Different Seasons

**DOI:** 10.3390/foods10092144

**Published:** 2021-09-10

**Authors:** Santiago P. Aubourg, Marcos Trigo, Ricardo Prego, Antonio Cobelo-García, Isabel Medina

**Affiliations:** 1 Department of Food Technology, Marine Research Institute (CSIC), c/E. Cabello 6, 36208 Vigo, Spain; mtrigo@iim.csic.es (M.T.); medina@iim.csic.es (I.M.); 2 Department of Oceanography, Marine Research Institute (CSIC), c/E. Cabello 6, 36208 Vigo, Spain; prego@iim.csic.es (R.P.); acobelo@iim.csic.es (A.C.-G.)

**Keywords:** *Doriteuthis gahi*, by-products, proximate composition, phospholipids, ω3 fatty acids, α-tocopherol, macrominerals, trace elements, nutritional value, human health

## Abstract

This study focuses on the extraction of nutritional and healthy constituents of whole by-products resulting from the commercialisation of Patagonian squid (*Doriteuthis gahi*). By-products corresponding to squid individuals captured at different seasons were comparatively analysed for proximate composition, lipid classes content, fatty acid (FA) profile, and macroelement and trace element composition. As a result, moisture, lipid, protein, and ash values were included in the ranges 829.0–842.8, 17.5–21.8, 106.0–123.7, and 9.3–13.3 g·kg^−1^ by-products, respectively. Phospholipids showed to be the most abundant lipid class (359.2–463.5 g·kg^−1^ lipids), while triacylglycerols were only present in a 9.5–13.1 g·kg^−1^ lipids range. Valuable levels were detected for α-tocopherol (539.6–973.3 mg·kg^−1^ lipids), polyunsaturated fatty acids (PUFA; 50.5–52.6 g·100 g^−1^ FA), ω3 PUFA (47.0–48.6 g·100 g^−1^ FA), PUFA/saturated FA ratio (1.4–1.6), and ω3/ω6 ratio (12.1–13.4). Among macroelements, *S*, *P*, and *Na* showed to be more abundant than *K*, *Mg*, and Ca. Profitable levels of *Co*, *Cu*, *Fe*, *Mn*, *Se*, and *Zn* were detected in all kinds of individuals. In spite of content variations found as a result of the capture season of Patagonian squid individuals, whole by-products of this cephalopod species can be considered as a profitable source to provide the food and pharmaceutical industries with useful value-added constituents.

## 1. Introduction

The wide chemical and biological diversity observed in marine fish and invertebrates make them an extraordinary source of highly valuable constituents that can be employed in many applications [1]. Among such constituents, highly nutritional and digestible proteins, lipid-soluble vitamins, essential minerals, and highly unsaturated fatty acids can be mentioned [2]. Notably, marine lipids are now the subject of a great deal of attention due to their high content of ω3 polyunsaturated fatty acids (PUFA), which have shown a positive role in preventing certain human diseases [3,4].

Marine species have shown substantial variations of chemical composition as a result of endogenous and exogenous effects [5]. Notably, the lipid matter has been recognised as the most highly affected. Concerning endogenous factors, marine species constituents have been shown to be inhomogeneously distributed along the body of a fish [6,7]. Related to exogenous effects, the capture season has shown to play a key role regarding temperature, feeding availability, and other factors in different types of marine species [8,9].

Fishing and aquaculture play an important role in human society’s development. Nowadays, annual seafood production contributes to over 170 million tonnes of fish and shellfish worldwide [10]. Nonetheless, only 50–60% of the total catch is used for direct human consumption, seafood processing being considered as one of the main sources of by-products (heads, blood, viscera, skin, tails, etc.) [11]. Hence, a large and considerable volume of undesired products is obtained, constituting an important source of environmental contamination unless efforts for their recovery are attained [12,13] and their commercial value can be enhanced via extraction of valuable constituents [14,15]. Remarkably, the highest concentration of high added value compounds such as minerals, lipids, amino acids, polysaccharides, and proteins is often in body parts of marine organisms that are commonly discarded [16,17].

Cephalopods represent a highly interesting biological group due to their nutritional value for human health and for their commercial significance [18,19]. Among cephalopod species, Patagonian squid (*Doriteuthis gahi* or *Loligo gahi*), a neritic species widely distributed along the Atlantic and Pacific coasts of South America, has attracted the attention of several countries as an important fishery resource [20]. Thus, the mantle tissue is commonly excised to be employed commercially, while the remaining parts of the body are considered as by-products and employed for meal production. Previous research on this species can be considered scarce, accounting for its employment for aquaculture feeding [21], collagen production [22] and detection of trace elements [23], and polycyclic aromatic hydrocarbons [24]. The present study focuses on the extraction of nutritional and healthy constituents from Patagonian squid by-products. In it, resulting by-products are pooled together and analysed as a single product for proximate composition, lipid fraction (classes and fatty acids), and essential element (macro elements and trace elements) content. The employment of whole by-products was considered useful on the basis of implying a fast and practical handling procedure. Additionally, the capture season effect on chemical composition was comparatively studied.

## 2. Materials and Methods

### 2.1. Starting Squid Material and Sampling Procedure

Fresh Patagonian squid (*Doriteuthis gahi*) by-products were provided by SERPESBA S.L.U. (Vigo, Spain). The squid was caught near the Argentinean coast in the southwest Atlantic Ocean in Austral summer (February), autumn (May), winter (August), and spring (November) of year 2020. In each case, squid samples were frozen (−40 °C) and transported to the Vigo (Spain) factory, where samples were thawed (overnight, 4 °C), the mantel being taken for commercialisation and the resulting by-products pooled together and transported in refrigerated conditions (4 °C) to our laboratory placed in the same town.

At each sampling time, 3.0 kg of whole squid by-products were divided into three different groups of 1.0 kg each that were analysed separately (*n* = 3, three replicates). In each group, squid by-products were homogenised before starting the different extraction procedures and analyses.

All solvents and chemical reagents used were of reagent grade (Merck, Darmstadt, Germany).

### 2.2. Proximate Analysis

Moisture was determined as the weight difference (1–2 g) before and after 4 h at 105 °C according to official method 950.46B [25]. Results were calculated as g·kg^−1^ squid by-products.

Lipids were extracted by the Bligh and Dyer [26] method, which employs a single-phase solubilisation of the lipids using chloroform–methanol (1:1) mixture. Results were calculated as g·kg^−1^ squid by-products.

Protein content was determined by the Kjeldahl method in agreement with procedure 928.08 (alternative II) [25]; a conversion factor of 6.25 was employed. Results were calculated as g·kg^−1^ squid by-products.

Ash was determined by heating at 550 °C according to the official method 920.153 [25]. Results were calculated as g·kg^−1^ squid by-products.

### 2.3. Lipid Class Analysis

The phospholipid (PL) content in the lipid extract was measured according to the method of Raheja et al. [27], which is based on formation of a complex with ammonium molybdate. Results were calculated as g PL·kg^−1^ crude lipid extracts.

The sterol (ST) content in the lipid extract was assessed by the method of Huang et al. [28], based on the Liebermann–Buchardt reaction. Results were calculated as g ST·kg^−1^ crude lipid extracts.

To evaluate the triacylglycerol (TG) presence, lipid extracts were first purified on 20 × 20 cm^2^ thin-layer chromatography plates coated with a 0.5 mm layer of silica gel G from Merck using a mixture of hexane-ethyl ether-acetic acid (90:10:1, v:v:v; two times) as eluent [29]. Once the TG fraction was purified, the method of Vioque and Holman [30] was used to measure the ester linkage content according to the conversion of the esters into hydroxamic acids and subsequent complexion with Fe (III). Results were calculated as g TG·kg^−1^ crude lipid extracts.

The free fatty acid (FFA) content in the lipid extract was determined following the method of Lowry and Tinsley [31], which is based on the formation of a complex with cupric acetate-pyridine. Results were calculated as g FFA·kg^−1^ crude lipid extracts.

The profile of tocopherol compounds was analysed according to the method of Cabrini et al. [32]. For this, squid by-products were extracted with hexane, which was eliminated under nitrogen flux. The resulting extracts were then dissolved in isopropanol and injected into an HPLC system (ODS column, 15 cm × 0.46 cm i.d.). The column was fluxed with methanol for 2 min; then, a gradient from 0 to 50% of isopropanol in 10 min was applied. Flow rate was 1.5 mL·min^−1^; detection was achieved at 280 nm. The possible presence of different tocopherol compounds (*α*, *β*, *γ* and *δ*) was checked, their content being calculated with calibration curves prepared from the corresponding commercial tocopherols and expressed as mg·kg^−1^ crude lipid extracts.

### 2.4. Analysis of the Fatty Acid Composition

Acid-catalysed esterification and transesterification by using acetyl chloride in methanol were employed for converting lipid extracts into fatty acid methyl ester (FAME). The resulting FAMEs were then analysed by gas–liquid chromatography (Perkin Elmer 8700 chromatograph, Madrid, Spain) [33]. Identification was carried out according to their retention times and comparison with standard mixtures (Qualmix Fish, Larodan, Malmo, Sweden; FAME Mix, Supelco, Inc., Bellefonte, PA, USA). For qualitative purposes, C19:0 fatty acid was used as internal standard, and peak areas were automatically integrated. The content of each fatty acid (FA) was expressed as g·100 g^−1^ of total FA.

Results concerning FA groups (saturated, SAT; monounsaturated, MUFA; PUFA; ω3 PUFA) and FA ratios (PUFA/SAT and ω3/ω6) were calculated.

### 2.5. Determination of Macroelements and Trace Elements

The content of six macroelements (*Ca*, *K*, *Mg*, *Na*, *P*, and *S*) and six trace elements (*Co*, *Cu*, *Fe*, *Mn*, *Se*, and *Zn*) was analysed according to the following procedure based on EPA 3050B [34] procedure. About 1 g of ground sample was put into a Teflon digestion flask with 9 mL of hyperpure 69% nitric acid (TMA), 3 mL of H_2_O_2_ (for ultratrace analysis) and 3 mL of Milli-Q water. The sample was then digested in a microwave oven (Ethos Easy Advance Microwave Digestion System, Milestone, Sorisole, Italy). After digestion treatment, samples were analysed by ICP-MS (inductively coupled plasma-mass spectrometry) using Agilent 7900 equipment (Agilent Technologies, Inc., Santa Clara, CA, USA). The quantification was carried out by external calibration with element standards traceable to NIST (National Institute of Standards and Technology) standards. The limits of detection were calculated with respect to the standard deviation of the blanks (LD = 3·SD blanks). Procedural blanks always accounted for <1% of element concentrations in the samples. Accuracy of the analytical procedures was ensured using DORM-2 certified reference material, prepared by the National Research Council of Canada (NRCC), as the quality control material.

Results obtained for the different elements were calculated as g·kg^−1^ squid by-products for macroelements and as mg·kg^−1^ squid by-products for trace elements, except for *Co* (μg·kg^−1^).

### 2.6. Statistical Analysis

Data (*n* = 3) obtained from the different chemical analyses were subjected to one-way ANOVA (*p* < 0.05) to investigate differences among the four different seasons (Statistica version 6.0, 2001; Statsoft Inc., Tulsa, OK, USA). Comparison of means was performed using a least-squares difference (LSD) method. Correlation values between proximate composition and minerals were also analysed.

## 3. Results and Discussion

### 3.1. Proximate Composition

Moisture showed to be the most abundant constituent in samples corresponding to all seasons, all values being included in the 829.0–842.8 g·kg^−1^ range (Table 1). According to average values, a progressive moisture increase was observed in squid samples following the sequence summer < autumn < winter < spring. Notably, spring by-products revealed significantly higher values (*p* < 0.05) than the remaining seasons. Lipid content (Table 1) showed to be included in the 17.5–21.8 g·kg^−1^ range. Comparison among capture times showed a similar increasing sequence for average values as in the case of moisture level. Thus, by-product samples from winter and spring showed higher levels (*p* < 0.05) than their counterparts from summer and autumn. Contrary to this direct relationship between moisture and lipid contents, edible parts of cephalopod species and marine species, in general, have shown an inverse relationship between both constituents [5,35].

Protein values were included in the 106.0–123.7 g·kg^−1^ range (Table 1). Comparison among current squid by-products obtained at different seasons provided a decreasing value from summer to spring so that samples corresponding to spring depicted the lowest proportion (*p* < 0.05) for this constituent. Therefore, an opposite trend to moisture and lipid values was concluded for proteins. Concerning the ash content, values were in all cases included in the 9.3–13.3 g·kg^−1^ range. A lower ash content (*p* < 0.05) was detected in samples corresponding to winter when compared with their counterpart by-products. However, a definite trend as a result of the season could not be concluded.

When compared with edible parts of cephalopod species, current data on proximate composition have shown higher moisture and lipid levels but lower protein content [5,35]; for ash level, values can be considered relatively similar to edible tissues of cephalopod species and marine ones in general [5]. Related to the current study, Kacem et al. [36] analysed the proximate composition of viscera fraction (stomach, intestines, and pyloric caeca) from *Sepia officinalis* captured in Tunisian coasts at different times (from October to May). As a result, similar values to the current study were obtained for moisture (75–85%), lipids (0.58–4.02%) and proteins (7.7–13.5%); however, substantially higher values were detected for ash (2.3–6.2%). Additionally, the highest levels for lipids and proteins were obtained in October, while moisture showed the highest levels in December and April.

Previous research related to non-edible parts of cephalopod species is focused on particular body parts considered as by-products. Thus, the squid (*Loligo formasana*) ovary showed a substantially different composition than the current whole by-product [19]; values for moisture, proteins, ash and lipids were, respectively, 72.1, 18.6, 1.4, and 0.5 expressed as g·100 g^−1^ tissue. Lipid content has been analysed in several studies in squid liver and gonad. Thus, Saito et al. [37] indicated a lipid content of 15.7–17.9% for liver and 1.0–1.4% for gonad in the case of Humboldt squid (*Dosidicus gigas*), while Hayashi and Kishimura [38] indicated higher lipid levels in the liver (56.9%) and a marked difference between ovary (13.9%) and testis (1.5%) levels in squid *Berryteuthis magister*. Hayashi [6] detected lipid contents in the ranges of 5.8–8.8% and 1.6–3.6% for liver and gonad in arrow squid (*Loligo bleekeri*), respectively.

### 3.2. Lipid Class Content

PL showed to be the most abundant lipid class (Table 2). Values were included in a 359.2–463.5 g·kg^−1^ range. On the basis of the average values obtained, a decreasing tendency was detected from winter to autumn; thus, such seasons depicted the highest and lowest (*p* < 0.05) values, respectively. PL have been described as having an important structural role in living bodies in general, being important constituents of cell membranes. Furthermore, and on the basis of their amphiphilic character, PL have recently attracted great attention as serving as drug delivery systems and having high bioavailability and protecting effect on different kinds of diseases [39,40]. Consequently, profitable functions related to pharmaceutical and food production industries have recently been developed for marine PL as having a high content on docosahexaenoic (DHA) and eicosapentaenoic (EPA) acids [41]. In this sense, current by-products, having a relatively high PL level, may provide an accurate source of marine PL.

ST levels showed straight differences as a result of the season, all values being included in the 115.0–132.1 g·kg^−1^ range (Table 2). Comparison among samples revealed a decreasing value (*p* < 0.05) from summer to spring. Therefore, a similar tendency is concluded for this lipid class than for protein content and opposite to water and lipid levels. However, an inverse ratio between sterol content and total crude lipids has been detected in edible parts of marine species [5,33]; this inverse ratio has been justified on the basis that ST are reported to contribute to functional properties and play structural roles in living bodies, as in the previously mentioned case of PL.

TG showed to be the less abundant lipid class from those analysed (Table 2). Values were included in all cases in the 9.5–13.1 g·kg^−1^ range. Comparison among samples corresponding to the different seasons indicated increasing average values from summer to spring; notably, TG levels in by-products from winter and spring were higher (*p* < 0.05) than in those corresponding to summer and autumn. Consequently, a similar trend for total lipid and moisture was detected, opposite to the trend of proteins and ST. Remarkably, a direct relationship between TG and total lipid levels has been proved in the edible parts of marine species, which agrees with the role of TG as being a storage or depot lipid class [5,33].

Compared with values detected in edible parts of cephalopod species and marine species in general [5,29], FFA levels of current by-products can be considered relatively high (Table 2). Such high values can be explained on the basis of the activity of lipases and phospholipases in visceral tissues [35,42]. Content on FFA would depend on the in vivo metabolic action of lipases and phospholipases on high-molecular-weight lipids (i.e., TG and PL), levels being especially higher in viscera than in edible tissues. Present FFA values were included in a wide range (i.e., 156.6–282.0 g·kg^−1^), showing a strong effect of season. Thus, comparison among average values provided an increasing tendency from winter to autumn, opposite to the tendency found for PL.

The qualitative analysis of the tocopherol fraction only revealed the presence in squid by-products of α-tocopherol, according to previous research on higher marine animals from natural diets [5]. Values for this endogenous antioxidant (i.e., vitamin E) were included in the 539.6–973.3 mg·kg^−1^ by-product range (Table 2). The highest levels (*p* < 0.05) were determined in summer and autumn samples, while the lowest (*p* < 0.05) were obtained in spring samples. On the basis of the average values, a decreasing tendency from summer to spring was detected. Consequently, a similar trend for protein and sterol content was concluded and opposite to moisture, crude lipid and TG levels. Values obtained were substantially higher than those observed in farmed (338–400 mg·kg^−1^ lipids) blackspot seabream (*Pagellus bogaraveo*) flesh but lower than in its counterpart wild fish (1327–1672 mg·kg^−1^ lipids) [29]. Furthermore, present α-tocopherol levels were higher than those determined in different muscle zones of megrim (9.8–14.1 mg·kg^−1^ lipids) (*Lepidorhombus whiffiagonis*) as well as in edible tissues of marine species in general [5]. Tocopherols are known lipid-soluble chain-breaking antioxidants, whose main role is to protect the unsaturated FA from oxidation [43]. Therefore, current squid by-products can be considered as a profitable and valuable source of α-tocopherol extraction.

Previous research accounts for studies on lipid class composition of single tissues considered as by-products from different squid species. Thus, Hayashi [6] detected TG as the most abundant lipid class in arrow squid (*Loligo bleekeri*) liver (45–63 g·100 g^−1^ lipids), while lipid classes such as PL, FFA, and ST showed lower values (0.4–0.7, 8.1–9.3, and 4.5–5.1 g·100 g^−1^ lipids, respectively). Furthermore, this author found low values for all the analysed lipid classes in the gonad (2.7–6.0, 0.2–0.3, 1.6–4.7, and 8.1–11.3 g·100 g^−1^ lipids in TG, PL, FFA, and ST, respectively). Later on, Hayashi and Kishimura [38] analysed the lipid classes of different tissues from squid *Berryteithis magister*. As a result, the liver showed a high TG presence (53 g·100 g^−1^ lipids), while FFA, ST, and PL provided a low content (8.2, 1.0, and 2.5 g·100 g^−1^ lipids, respectively); contrary to the liver, only the gonad revealed a trace presence of TG, while FFA, ST, and PL depicted higher values in the ovary (5.8, 39.9, and 19.4 g·100 g^−1^ lipids, respectively) and in testis (27.1, 17.6, and 55.1 g·100 g^−1^ lipids, respectively). Lipid classes composition of the liver and gonad was analysed in Humboldt squid (*Dosidicus gigas*) by Saito et al. [37]. According to the above-mentioned studies, TG showed to be the most abundant lipid class in the liver (21–27 g·100 g^−1^ lipids); meanwhile, values for ST, FFA, phosphatidyl-ethanolamine (PE), and phosphatidyl-choline (PC) in the liver were 14–16, 26, 5–6, and 0.7–24 g·100 g^−1^ lipids, respectively. Concerning lipid class composition of the gonad, TG showed to be the less abundant class (2.4–3.6 g·100 g^−1^ lipids), and FFA reflected lower levels than in the liver (14–17 g·100 g^−1^ lipids); however, ST, PE, and PC provided higher values than in the liver (19–22, 13–19, and 25–28 g·100 g^−1^ lipids, respectively).

### 3.3. Fatty Acid Composition

The qualitative analysis of FA composition of squid by-products can be considered very similar to that of the edible part of cephalopod species and marine species in general, showing a wide variety of saturated, monounsaturated, and polyunsaturated FA (Table 3) [5,33,35]. From a quantitative point of view, the most abundant FA was DHA, followed by C16:0 and EPA. It is worth pointing out the high presence of DHA (29.5–30.8 g·100 g^−1^ range) and EPA (15.9–17.2 g·100 g^−1^ range). Other FAs present in a substantial proportion were C20:1ω9, C18:0, C18:1ω9, C14:0, C20:4ω6, and C18:1ω7.

**Figure 1:**
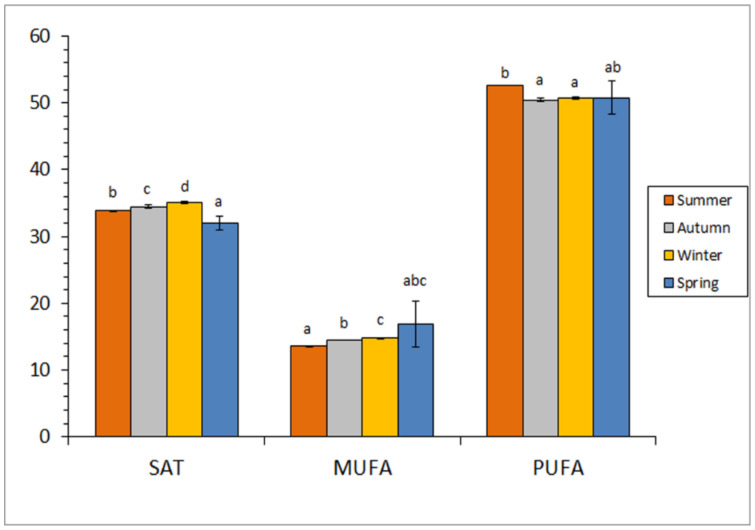
Fatty acid (FA) group analysis of by-products from squid captured at different seasons. Abbreviations employed: SAT (saturated FA), MUFA (monounsaturated FA), and PUFA (polyunsaturated FA). Data expressed as g·100 g^−1^ total FA. Average values of three independent determinations (*n* = 3); standard deviations are indicated by bars. For each FA group, different low-case letters (a, b, c, d) denote significant differences (*p* < 0.05) as a result of season.

The analysis of FA groups indicated that the PUFA group was the most abundant (50.5–52.6 g·100 g^−1^ range; Figure 1) on the basis of the above-mentioned high levels for DHA and EPA (Table 3). Comparison among samples corresponding to the different seasons showed an increasing average value for the PUFA group from autumn to summer, values from summer samples being higher (*p* < 0.05) than their counterparts from winter and autumn (Figure 1). The SAT group showed values included in the 32.0–35.1 g·100 g^−1^ range (Figure 1). This group provided the following increasing tendency (*p* < 0.05): spring < summer < autumn < winter.

Finally, MUFA showed to be the less abundant group, values being included in the 13.6–16.9 g·100 g^−1^ range (Figure 1). Average MUFA values revealed an increasing content when taking into account by-products from summer to spring. Differences were found significant (*p* < 0.05) by comparison of samples corresponding to summer, autumn, and winter.

According to recent studies [3,4], the total content of ω3 unsaturated FA is being considered a highly valuable parameter. As a consequence of the high presence of DHA and EPA, high ω3 scores were detected in all kinds of the current samples (47.0–48.6 g·100 g^−1^) (Table 3). A decreasing average value was detected from summer to spring, differences between summer by-products and their counterparts from autumn and winter being significant (*p* < 0.05).

**Figure 2:**
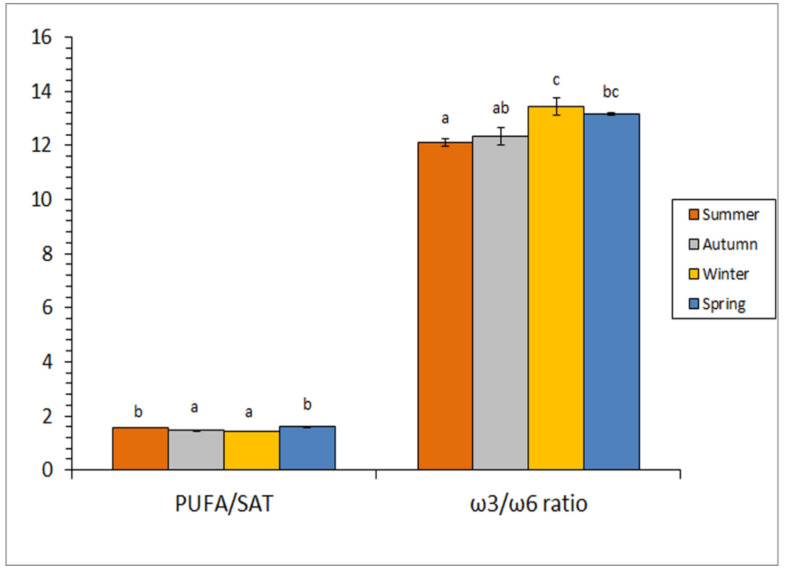
Fatty acid (FA) group analysis of by-products from squid captured at different seasons. Abbreviations employed: PUFA (polyunsaturated FA) and SAT (saturated FA). Average values of three independent determinations (*n* = 3); standard deviations are indicated by bars. For each FA ratio, different low-case letters (a, b, c) denote significant differences (*p* < 0.05) as a result of season.

Great attention has been accorded to some FA ratios on the basis of their possible effect on nutritional value and human health. One of such ratios is PUFA/SAT. In the current study, values were included in the 1.4–1.6 range (Figure 2). Considering the average values, a decreasing tendency from spring to winter was detected for this ratio. Notably, values for spring and summer by-products were higher (*p* < 0.05) than their counterparts from autumn and winter.

It is recognised nowadays that most Western countries do not consume adequate levels of ω3 FA. Thus, great attention has been paid to the ω3/ω6 ratio of foods included in the human diet [44,45]. In order to prevent inflammatory, cardiovascular, and neurological disorders, the World Health Organization (WHO) currently recommends that this ratio should not be below 1:10 in the human diet [46]. Additionally, the European Nutritional Society reported that a human diet with an ω3/ω6 ratio of 1:5 or higher would have health benefits [47]. In the present study, a 12.1–13.4 ratio range was observed (Figure 2); this value is similar to the one obtained for edible tissues of wild fish species such as black spot seabream (*P. bogaraveo* [29]) and megrim (*L. whiffiagonis*) [33]. Notably, the present highest average values were obtained in winter samples, while the lowest ones were detected in by-products corresponding to summer. Therefore, current ω3/ω6 ratios obtained for squid by-products can be considered as profitable and highly valuable from a health point of view.

Previous research accounts for information on FA composition related to cephalopod species tissues considered as by-products after excision of the main commercial part of the body. Hayashi [6] analysed the FA composition of the liver and gonad of arrow squid (*Loligo bleekeri*). Thus, qualitative FA composition was very similar to the current study (i.e., C:16:0, DHA, and EPA as the most abundant FA). Additionally, in agreement with the current research, PUFA showed to be the most abundant FA group in both liver and gonad (36–39 and 38–44%, respectively), while MUFA was less abundant (25–29% and 19–20%, respectively).

A similar FA distribution was also found in the ovary and liver of common octopus (*Octopus vulgaris*), DHA, C16:0, and EPA being the most abundant FA [35]. Furthermore, the PUFA group was major in both tissues (49–56% and 46–52%, respectively), while SAT group was less abundant (12–14% and 22–25%, respectively). Additionally, the authors calculated an ω3/ω6 ratio of 4.2–8.3 for the ovary and of 5.0–9.1 for the liver.

A different FA distribution was detected by Shen et al. [48] in cuttlefish (*Sepiella maindroni de Rochebrum*) viscera when compared with the current results. As a result, the most abundant FA were C18:1ω9, DHA, and C20:1ω11, the MUFA group being the most abundant (50%) and SAT less (19%). Kacem et al. [38] also detected a different distribution of FA in *Sepia officinalis* viscera (i.e., stomach, intestines, and pyloric caeca). Thus, the most abundant FA were C16:0, EPA, and C18:0; furthermore, an equilibrated FA group distribution (SAT, MUFA, and PUFA) was detected (29–43%, 22–25%, and 29–44%, respectively). Notably, a lower total ω3 content than in the current study was obtained (21–26%).

Recently, a similar FA group distribution than in the present research was observed by Singh et al. [19] in squid (*Loligo formasana*) ovary. Values obtained for SAT, MUFA, and PUFA groups were 39.4%, 12.9%, and 43.8%, respectively. The most abundant FA were DHA, EPA, and C20:4ω6. However, substantially lower values were detected for total ω3 FA (37.3%) and ω3/ω6 ratio (2.1) than in the current research.

### 3.4. Content of Macroelements and Trace Elements

Content (g·kg^−1^ by-products) on all by-product macroelements provided important variations according to the capture season considered (Table 4); this effect was especially notorious for *Ca* (0.4–1.1), *K* (0.9–1.2), and *Na* (1.7–2.6). In most cases, the lowest average values were obtained in samples corresponding to winter and spring. Thus, the winter season revealed the lowest values (*p* < 0.05) for *Ca*, *Mg*, and *Na*; meanwhile, the lowest average values for *K*, *P*, and *S* were found in individuals corresponding to spring, differences being significant (*p* < 0.05) only in the case of *K*. Contrary, the summer season provided the highest average values for *K*, *Mg*, *P*, and *S*, differences being significant (*p* < 0.05) for *K*, *P*, and *S*. Finally, squid by-products corresponding to autumn revealed the highest scores (*p* < 0.05) for *Ca* and *Na*.

Season differences were also detected for all trace elements analysed in the current squid by-products (Table 4). Differences were substantially important in *Fe* (5.8–20.4 mg·kg^−1^), *Se* (0.9–1.4 mg·kg^−1^), and *Co* (5.5–8.4 μg·kg^−1^), while *Cu* (49.0–60.0 mg·kg^−1^), *Mn* (0.58–0.67 mg·kg^−1^), and *Zn* (19.3–25.1 mg·kg^−1^) provided shorter variations. According to macroelement season distribution, winter individuals revealed the lowest average values for most of the trace elements analysed, such as *Fe*, *Mn*, *Zn*, and *Co*; differences were found to be significant (*p* < 0.05) for *Fe*, *Zn*, and *Co*. Notably, the lowest values (*p* < 0.05) for *Cu* and *Se* were detected in squid by-products corresponding to the spring season, while *Fe*, *Mn*, and *Co* showed the highest average values at this season time. *Se* and *Zn* depicted the highest values (*p* < 0.05) in summer and autumn by-products.

Living in a mineral-rich medium, marine organisms accumulate macroelements and trace elements from diet and the aquatic medium and incorporate them in their tissues and organs so as to be considered a good source of essential elements [49,50]. Among trace elements, *Cu*, *Se*, *Mn*, and *Zn* are contained in enzymes that protect cells against oxidant stress and, therefore, may be considered biological antioxidants [51]. Interestingly, *Se* has been reported to be a health-promoting ingredient in foods because of a wide number of important biological functions being related to the activity of certain Se-containing proteins [52]. Previous research concerning the presence of macroelements and trace elements in squid tissues has been especially concentrated on edible parts. Thus, the following average values have been reported [5]: 1–3 g·kg^−1^ tissue (*K*, *P*, *S*, and *Na*), 0.1–1 g·kg^−1^ tissue (*Ca* and *Mg*), 2–100 mg·kg^−1^ tissue (*Fe*, *Zn*, and *Cu*), 0.2–4 mg·kg^−1^ tissue (*Mn*), 0.01–1 mg·kg^−1^ tissue (*Co*). In the case of *Se*, 0.10–0.45 mg·kg^−1^ tissue has been detected for edible parts of cephalopod species [50]. Comparison with the current data obtained proves that present squid by-products can be considered as a valuable source of most of the macroelements and trace elements determined. This could be especially interesting in the case of *Se* presence, while *Co* content could be considered relatively low.

Macroelements and trace elements corresponding to the transition and electronegative groups of the Periodic Table have been reported to be strongly bound to other tissue constituents in seafood [5,49]. Therefore, the relationship between element presence and moisture, lipid, protein, and ash content was investigated in the present work. For it, correlation values were studied. Concerning macroelements, good correlation values were detected for *K* (*r* = −0.89, −0.86, and −0.93), *P* (*r* = −0.83, −0.92, and 0.85), and *S* (*r* = 0.93, 0.94, and 0.90) with moisture, lipid, and protein content, respectively. Meanwhile, ash level provided accurate correlation values with *Ca* (*r* = 0.78), *Mg* (*r* = 0.83), and *Na* (*r* = 0.91). Related to trace elements, the best correlation values were detected for *Se* (*r* = −0.94 and −0.91) and *Zn* (*r* = −0.80 and −0.91) with moisture and crude lipid levels, respectively.

Previous research concerning the concentration of macroelements and trace elements of inedible cephalopod parts has been concentrated on the liver (i.e., digestive gland or hepatopancreas), according to its known role of bioaccumulation. Related to the current squid species, Falandysz [23] analysed the content of several trace elements in different edible and inedible tissues (raw skinless mantle, raw arms and crone, raw fin, and raw whole squid). Values obtained (mg·kg^−1^ tissue) were included in the following ranges: 5.5–22 (*Cu*), 12–16 (*Zn*), 0.38–0.48 (*Mn*), and 2.2–3.4 (*Fe*). Comparison with current data (Table 4) shows that higher scores were detected in the current study.

Concerning other squid species by-products, Falandysz [53] analysed the macroelement (*Na*, *K*, *Ca*, and *Mg*) content in edible parts, as well as in inedible tissues (i.e., liver and intestines and head) of raw *Loligo opalescens.* Edible parts revealed the lowest presence on all macroelements. Notably, levels (g·kg^−1^ tissue) for liver showed to be higher in the case of *Na* (5.0–51) and *K* (7.2–18) when compared with intestines and head values (2.0–3.1 and 1.8–4.1, respectively). In the case of *Ca* and *Mg*, relatively similar values were detected (74–200 and 280–650 for liver, respectively; 42–110 and 380–520 for intestines and head, respectively) in both inedible tissues.

Storelli et al. [54] studied the *Cu*, *Zn*, and *Se* presence in the flesh and hepatopancreas of *Illex coindeti* and *Loligo vulgaris* from the Mediterranean Sea. The hepatopancreas showed higher element concentrations than flesh. Thus, values (µg·g^−1^ tissue) for *Cu*, *Zn*, and *Se* were 11.0, 36.4, and 1.2 (flesh) and 119.0, 145.0, and 2.0 (hepatopancreas), respectively.

The presence of *Cu* and *Zn* was analysed in different tissues of European squid (*Loligo vulgaris*) [55] and in flying squid (*Sthenoteuthis oualaniensis*) [56]. In both studies, the liver showed higher levels of both trace elements than edible parts (arm and mantle), highlighting its major role in their bioaccumulation and concentration. Thus, values (μg·g^−1^ dry weight) detected in European squid liver were 150–250 and 100–200, respectively, for both elements, while flying squid liver revealed levels of 20.2–358.2 and 50.3–186.7, respectively.

## 4. Conclusions

The present research provides the first approach on the chemical composition of squid Patagonian by-products in order to be used as a valuable source of nutritional and healthy constituents for the food and pharmaceutical industries. Compared with edible cephalopod tissues in general, results showed a higher level of moisture and crude lipids but lowered proteins. Remarkably, highly profitable values were detected for PL, α-tocopherol, DHA, EPA, and total ω3 FA content. Furthermore, highly healthy values for PUFA/SAT and ω3/ω6 ratios were detected. Among macroelements, *S*, *P*, and *Na* showed to be more abundant than *K*, *Mg*, and *Ca*, according to general data on edible portions of marine species. Profitable levels of essential trace elements (*Cu*, *Fe*, *Mn*, *Se*, and *Zn*) were detected in all kinds of individuals, especially in the case of *Se*.

The seasonal study revealed a marked effect of capture time on chemical composition. In spite of the varying distribution of most chemical constituents throughout the different seasons, resulting by-products from Patagonian squid can be considered in all cases as a valuable source of nutritional and healthy constituents for the human diet. Results presented in this work constitute a promising basis in order to apply whole by-products from marine species in general to obtain bioactive compounds, showing the advantages of not having to previously separate particular by-product tissues (i.e., hepatopancreas, gonad, etc.) and implying a simplified handling procedure. The current study on by-products employment reinforces the development of green strategies focused on the extraction of added-value constituents on the enhancement of their economic value and on reducing the environmental drawbacks resulting from their accumulation.

As for edible parts of seafood in general, proper handling and storage during the processing of Patagonic squid by-products ought to be developed to avoid damage mechanisms such as lipid oxidation and microbial proliferation. Furthermore, to prevent health-associated risks (toxic elements, especially), by-products composition ought to agree with international regulation before practical and commercial employments are developed.

## Figures and Tables

**Table 1:** Proximate composition (g·kg^−1^) of by-products from squid captured at different seasons *.

Chemical Constituent	Capture Season			
	Summer	Autumn	Winter	Spring
Moisture	829.0 a (5.0)	831.6 a (3.8)	837.5 b (0.8)	842.8 c (3.5)
Lipids	17.5 a (0.4)	19.2 b (0.5)	21.5 c (0.3)	21.8 c (0.7)
Proteins	123.7 c (4.0)	118.1 bc (3.4)	114.0 b (1.7)	106.0 a (5.2)
Ash	12.1 b (0.4)	13.3 b (1.3)	9.3 a (0.1)	12.7 b (0.3)

* Average values of three independent determinations (*n* = 3); standard deviations are indicated in brackets. Average values followed by different low-case letters denote significant differences (*p* < 0.05) as a result of season.

**Table 2:** Lipid composition * of by-products from squid captured at different seasons **.

Lipid Class	Capture Season			
	Summer	Autumn	Winter	Spring
Phospholipids	398.2 b (18.1)	359.2 a (10.3)	463.5 c (6.4)	412.6 b (11.7)
Sterols	132.1 d (2.2)	127.2 c (1.4)	122.1 b (1.8)	115.0 a (1.8)
Triacylglycerols	9.5 a (0.7)	10.2 a (0.3)	12.9 b (0.4)	13.1 b (0.5)
Free fatty acids	274.3 c (3.8)	282.0 c (13.5)	156.6 a (1.2)	242.4 b (8.6)
Alpha-tocopherol	973.3 c (28.0)	949.7 c (65.9)	617.6 b (21.0)	539.6 a (17.5)

* Data expressed as g·kg^−1^ crude lipid extract, except for alpha-tocopherol (mg·kg^−1^ lipids). ** Average values of three independent determinations (*n* = 3); standard deviations are indicated in brackets. Average values followed by different low-case letters denote significant differences (*p* < 0.05) as a result of season.

**Table 3:** Fatty acid (FA) analysis of by-products from squid captured at different seasons *.

FA	Capture Season			
	Summer	Autumn	Winter	Spring
14:0	3.01 a (0.09)	3.24 b (0.11)	3.33 b (0.02)	3.80 c (0.15)
15:0	0.58 b (0.00)	0.62 c (0.01)	0.44 a (0.02)	0.52 b (0.04)
16:0	24.35 a (0.02)	24.71 b (0.06)	26.16 d (0.17)	25.74 c (0.05)
16:1ω7	1.31 a (0.00)	1.59 b (0.07)	1.57 b (0.02)	1.64 ab (0.28)
17:0	1.34 bc (0.00)	1.42 c (0.05)	1.03 a (0.01)	1.24 b (0.08)
18:0	4.61 b (0.02)	4.54 b (0.08)	4.22 a (0.02)	4.25 a (0.01)
18:1ω9	3.63 a (0.03)	4.14 bc (0.05)	3.95 b (0.02)	4.33 c (0.28)
18:1ω7	2.05 a (0.04)	2.15 ab (0.04)	2.17 b (0.02)	2.23 b (0.04)
18:2ω6	0.57 b (0.02)	0.57 b (0.01)	0.47 a (0.02)	0.59 b (0.05)
20:1ω9	5.30 a (0.02)	5.54 b (0.06)	5.41 a (0.02)	5.27 a (0.12)
20:2ω6	0.78 c (0.02)	0.58 b (0.05)	0.37 a (0.04)	0.39 a (0.02)
20:4ω6	2.43 b (0.03)	2.42 ab (0.04)	2.41 ab (0.03)	2.30 a (0.05)
22:1ω9	0.57 a (0.01)	0.58 a (0.01)	0.58 a (0.03)	0.58 a (0.02)
20:5ω3	17.24 c (0.03)	16.89 b (0.10)	15.93 a (0.12)	16.18 a (0.10)
22:4ω6	0.23 a (0.03)	0.24 a (0.00)	0.24 a (0.03)	0.22 a (0.04)
24:1ω9	0.69 a (0.02)	0.75 a (0.04)	0.71 a (0.02)	0.69 a (0.02)
22:5ω3	0.51 a (0.04)	0.50 a (0.02)	0.51 a (0.08)	0.53 a (0.01)
22:6ω3	30.79 c (0.03)	29.52 a (0.35)	30.48 b (0.19)	29.45 ab (0.79)
Total ω3	48.59 b (0.01)	47.05 a (0.23)	46.97 a (0.24)	46.11 ab (1.37)

* Data expressed as g·100 g^−1^ of total FA. Average values of three independent determinations (*n* = 3); standard deviations are indicated in brackets. Average values followed by different low-case letters denote significant differences (*p* < 0.05) as a result of season.

**Table 4:** Content on macroelements and trace elements * of by-products from squid captured at different seasons **.

	Capture Season			
	Summer	Autumn	Winter	Spring
Macroelement				
*Ca*	0.686 b (0.017)	0.831 c (0.064)	0.399 a (0.029)	1.131 d (0.042)
*K*	1.239 c (0.027)	1.071 b (0.038)	1.039 b (0.010)	0.881 a (0.020)
*Mg*	0.503 c (0.015)	0.487 bc (0.002)	0.398 a (0.002)	0.469 b (0.009)
*Na*	2.406 b (0.040)	2.572 c (0.028)	1.685 a (0.025)	2.276 b (0.114)
*P*	2.646 c (0.084)	2.180 b (0.069)	1.970 a (0.025)	1.913 a (0.082)
*S*	3.303 c (0.010)	3.151 b (0.036)	2.811 a (0.014)	2.704 a (0.084)
Trace element				
*Cu*	55.51 b (2.01)	59.24 bc (3.35)	59.97 c (0.51)	48.89 a (3.97)
*Fe*	9.31 b (1.49)	11.43 c (1.90)	5.79 a (1.08)	20.43 c (6.07)
*Mn*	0.637 ab (0.036)	0.595 ab (0.032)	0.581 a (0.025)	0.668 b (0.051)
*Se*	1.354 c (0.109)	1.303 c (0.018)	1.032 b (0.004)	0.888 a (0.020)
*Zn*	25.09 b (2.46)	23.86 b (0.54)	19.27 a (0.54)	20.62 a (1.51)
*Co*	6.85 b (0.53)	7.37 b (0.58)	5.45 a (0.11)	8.35 b (0.86)

* Data expressed as g·kg^−1^ by-products (macroelements) and mg·kg^−1^ by-products (microelements), except for *Co* (μg·kg^−1^ by-products). ** Average values of three independent determinations (*n* = 3); standard deviations are indicated in brackets. Average values followed by different low-case letters denote significant differences (*p* < 0.05) as a result of season.

## Data Availability

The data presented in this study are available on request from the corresponding author.
